# Were there any missing mediators between visual impairment and anxiety symptoms? Results from Chinese Longitudinal Healthy Longevity Survey

**DOI:** 10.3389/fpubh.2024.1448638

**Published:** 2024-10-16

**Authors:** Wen Yao, Yuan Cao, Yuan Tian, Yuanyuan Liu, Xin Hua, Fang Chen

**Affiliations:** ^1^Department of Ophthalmology, Northern Jiangsu People’s Hospital, Yangzhou, China; ^2^Department of Cardiology, Jinshan Hospital of Fudan University, Shanghai, China

**Keywords:** visual impairment, anxiety, sleep, exercise, diet, mediating role

## Abstract

**Introduction:**

Visual impairment, encompassing low visual acuity and visual field loss, significantly impacts the older adult population worldwide, leading to increased disability and mortality risks. Recent studies suggest a strong association between visual impairment and anxiety, particularly among older adults. This study aims to explore the relationship between visual impairment and anxiety symptoms in older adult individuals in China, and to investigate potential mediating factors.

**Methods:**

Data for this study were derived from the 2018 Chinese Longitudinal Healthy Longevity Survey (CLHLS), including 11,702 participants aged 65 and older. Visual impairment was assessed through self-reported visual function, while anxiety symptoms were measured using the 7-item Generalized Anxiety Disorder scale (GAD-7). Additional assessments included sleep quality and duration, exercise status, and dietary diversity. Logistic regression models and mediation analysis were employed to explore associations and mediating effects.

**Results:**

The findings indicate that visual impairment is significantly associated with increased anxiety symptoms among the older adult (OR = 1.51, 95% CI: 1.32–1.72, *p* < 0.001). Mediation analysis revealed that sleep quality, dietary diversity score (DDS), and plant-based DDS significantly mediated the relationship between visual impairment and anxiety. In contrast, sleep duration, exercise, and animal-based DDS did not show significant mediating effects.

**Conclusion:**

Visual impairment is a crucial predictor of anxiety symptoms in the older adult. Improving sleep quality and promoting a diverse plant-based diet may mitigate anxiety symptoms in this population. Interventions targeting these areas could enhance the mental health and quality of life of older adult individuals with visual impairment.

## 1 Introduction

Visual impairment which involves low visual acuity or visual field loss, affects billions of older adults worldwide and represents a significant cause of disability. By 2050, it is projected that the older adult will make up nearly one-quarter of the global population (1). The prevalence and incidence of visual impairment increase with age (2). Currently, China is among the countries with the highest number of people suffering from vision impairment globally. Visual impairment not only severely affects patients’ visual function and quality of life but also increases their risk of mortality. Recent epidemiological studies have indicated that older adults with visual impairment or blindness have 1.8–2.9 times greater risks of anxiety disorders worldwide (3). Kempen et al. reported that the prevalence of anxiety in older adults with visual impairment is twice as high as in those without (4). Given that pathological Visual impairment is usually considered an irreversible, progressive disease, patients often experience anxiety (5, 6). The presence of anxiety generally predicts worse outcomes for chronic physical health conditions and frequently co-occurs with major depressive disorder (7). Therefore, it is crucial to investigate the association between visual disabilities and anxiety, and to understand the underlying risk factors for anxiety prevention.

Anxiety is a common adverse psychological state among older adult patients with chronic diseases, severely impacting their mental health and quality of life (8). However, anxiety, characterized by persistent feelings of fear and worry that interfere with daily life, is highly prevalent in patients with visual impairment, ranging from 9.6 to 30% (9), but often neglected or not diagnosed in a timely manner. A population-based cohort study of over 34,000 people found that visual impairment was associated with a two-fold increase in anxiety (10).

Several potential mediators have been identified in the relationship between visual impairment and anxiety. These factors include the financial burden of frequent healthcare visits, difficulties in performing basic self-care activities, and uncertainty regarding the prognosis of ocular diseases (11). Visual impairments often correlate with economic disadvantage, as evidenced by higher rates of visual impairment in low-income countries (12). This economic strain can exacerbate anxiety, as individuals with visual impairments may struggle with additional costs for medical care, adaptive devices, and support services, further stressing their financial resources. Moreover, the challenges in self-care due to visual impairment can significantly contribute to anxiety (13). The inability to perform routine tasks independently can lead to feelings of helplessness and lower self-esteem, both of which are known contributors to anxiety. The uncertainty regarding the progression and prognosis of visual diseases adds another layer of psychological stress, as individuals may constantly worry about the potential worsening of their condition and its implications for their future independence and quality of life (14).

Despite these studies, there remains a scarcity of studies exploring whether poor visual impairment is an independent predictor of anxiety or if the impact of reduced visual acuity on anxiety outcomes is indirectly mediated by health and social factors. Studies on the associations between visual impairment and anxiety have not fully focused on older adults. The existing literature is limited by small sample sizes and short follow-up periods. Few studies have assessed the association between combined visual impairment and anxiety. Therefore, we first explored the association between visual impairment and anxiety symptoms in middle-aged and older adult individuals in our study. Additionally, we further investigated the potential mediating effects that may exist. We hope to identify possible moderating factors through this research, which could help alleviate anxiety in Chinese older adult individuals with visual impairment.

## 2 Method

### 2.1 Study population

**Figure 1:**
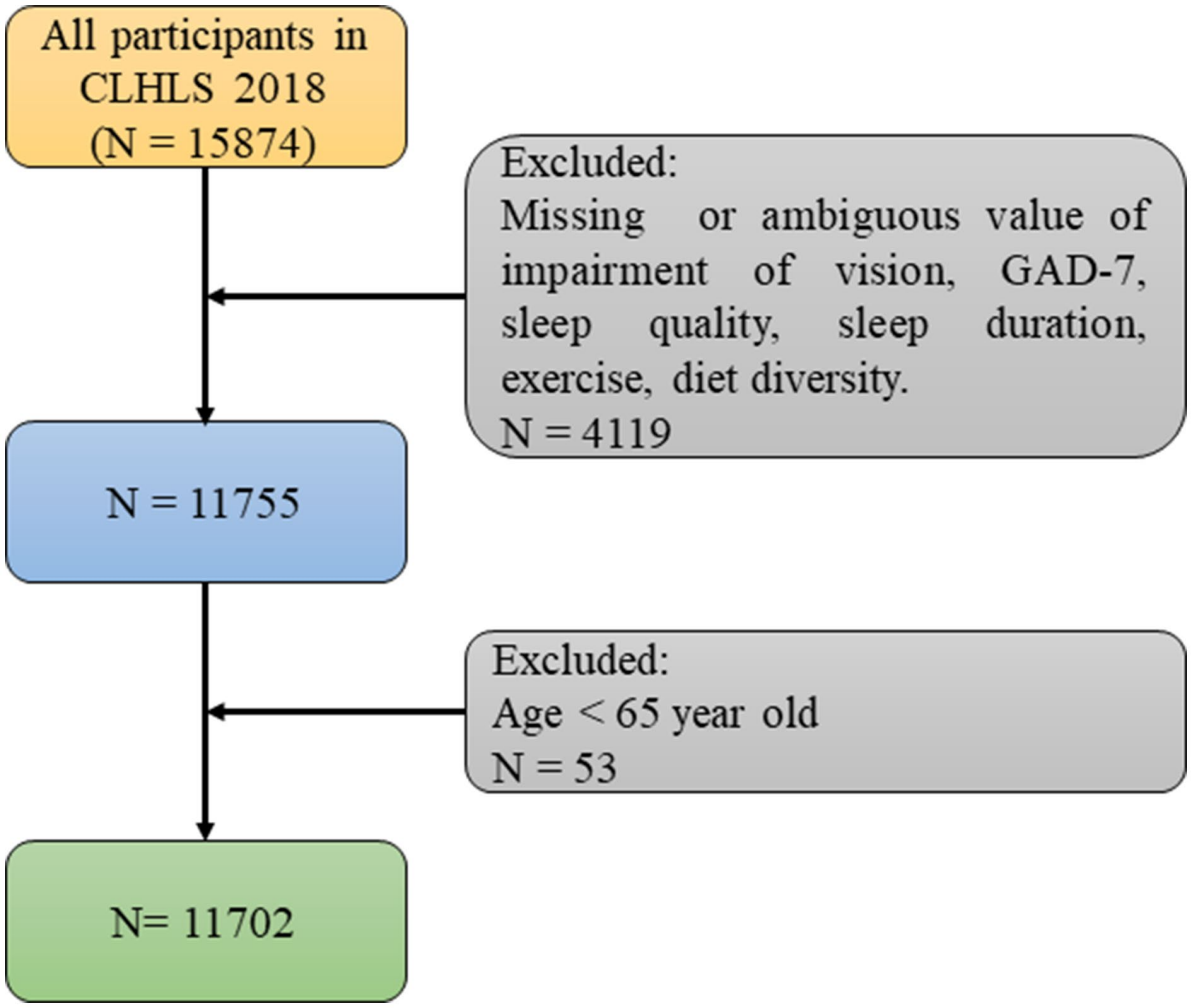
Flow chart of how to select participants for this study.

Our study adopts a cross-sectional observational design, analyzing data collected at a specific point in time (2017–2018) to examine the association between visual impairment and anxiety symptoms among older adult individuals, as well as potential mediator. With regard to study duration, we conduct this study between conducted over a three-month period from April to June 2024. The data used in this article was obtained from the 2018 dataset of the Chinese Longitudinal Healthy Longevity Survey (CLHLS) published by Peking University. The survey covered a total of 23 provinces and regions, accounting for approximately 85% of the total area of China. The questionnaire used in this study included information about demographic and sociological characteristics, family background, economic status, living conditions and health status. Individuals with missing or ambiguous data related to vision impairment, GAD-7 scores, sleep quality, sleep duration, exercise, and dietary diversity were excluded. Individuals under the age of 65 were also excluded. For other covariates with missing values, the k-nearest neighbors (KNN) imputation method was used to fill in the missing values (Figure 1). The CLHLS obtained approval from the Campus Institutional Review Board of Duke University (Pro00062871) and the Biomedical Ethics Committee of Peking University (IRB00001052-13074). Informed consent was obtained from all participants during the interviews.

### 2.2 Assessment of visual impairment

Visual impairment was evaluated using the following question, “Visual function: can you see the break in the circle?” This question included four levels: 1 = “can see and distinguish,” 2 = “can see only,” 3 = “cannot see,” and 4 = “blind.” For statistical convenience, the levels of “can see only,” “cannot see,” and “blind” were all classified as visual impairment (15–17).

### 2.3 Assessment of anxiety symptoms

The 7-item Generalized Anxiety Disorder scale (GAD-7) was used to assess anxiety symptoms in the CLHLS (18). This scale is widely used in China to evaluate anxiety for the older adult. Participants were asked to respond to questions based on their feelings over the past 2 weeks. Each question included four levels: 0 = never, 1 = for several days, 2 = more than half of the days, and 3 = almost every day. Anxiety scores for each Participants ranged from 0 to 21. Participants with scores ≥5 were identified as having anxiety symptoms in this study. Higher scores indicated more severe anxiety. The internal consistency reliability of the anxiety scale was satisfactory (Cronbach’s alpha coefficient = 0.920).

### 2.4 Assessment of sleep quality and sleep duration

Sleep quality was assessed using the question: “How is your sleep quality now.” In this question, sleep quality was categorized into five levels, including “very good,” “good,” “fair,” “poor,” and “very poor.” For further statistical analysis, “very good” and “good” were classified as good, while “poor” and “very poor” were classified as poor (19–21). Sleep duration was assessed based on the question, “How many hours do you usually sleep each day?” Sleep duration was analyzed as a continuous variable in this study (22).

### 2.5 Assessment of exercise status

According to the CLHLS questionnaire, exercise status was evaluated using the question, “Do you exercise at present” (23). This included two levels: 1 = “yes,” 2 = “no.”

### 2.6 Dietary diversity score

To evaluate nutritional diversity, we developed a 10-point dietary diversity score (DDS) in this study (24–26). Using a food-taking-frequency questionnaire covering vegetables, fruits, legumes, nuts, garlic, mushrooms, meat, eggs, fish and dairy products, we assigned 10-point scale for regular or almost daily consumption of each food category, with 0 points for other cases. The total DDS for each participant was calculated based on this scoring system, ranging from 0 to 10. Additionally, we assessed animal-based DDS and plant-based DDS. Animal DDS tracked the frequency of consumption of meat, fish, eggs, and dairy products, with scores ranging from 0 to 4. Plant DDS tracked the frequency of consumption of vegetables, fruits, legumes, garlic, nuts, and mushrooms—with scores ranging from 0 to 6. This scoring system allowed us to elucidate the mediating role of dietary diversity in the relationship between visual impairment and anxiety, and further to examine the roles of plant-based and animal-based dietary diversity separately.

### 2.7 Covariates

Based on previous research, we identified relevant variables that could potentially impact the study outcomes. Demographic covariates included age (65–80, 80–100, over 100) and gender (male, female). Socioeconomic covariates included residence (“city,” “town” and “rural”), marital status (“married” and “unmarried”), educational level (illiteracy, primary school, middle school, and above), medical service (yes, no), and annual household income (based on the annual household income at the time of the interview, categorized as greater than 100,000 RMB and less than or equal to 100,000 RMB). Health behavior variables included smoking status (“non-smoker” and “smoker”), drinking status (“non-drinker” and “drinker”), body mass index (BMI), hypertension (yes, no), diabetes (yes, no), heart disease (yes, no), stroke (yes, no) and cancer (yes, no). Depressive symptoms were assessed using the Center for Epidemiologic Studies Depression Scale (CES-D) (27). Responses were categorized as: “rarely,” “sometimes” (1–2 days), “occasionally” (3–4 days), and “always” (5–7 days). Within the scale, two positively worded items, “I was happy” and “I felt hopeful about the future,” were reverse coded. All responses were then coded from 0 to 3, corresponding to “rarely” to “always.” The sleep quality item from the CES-D-10 was absent in the CLHLS. Therefore, in this study, the CES-D-9 total score ranges from 0 to 27, with higher scores indicating more severe depressive symptoms. The Cronbach’s alpha for the CES-D-9 in this study was 0.706, indicating acceptable internal consistency. A score of ≥9 was used to identify individuals with significant depressive symptoms (20).

### 2.8 Data analysis

The basic characteristics of participants were reported as mean (SD) for continuous variables and number (percentage) for categorical variables. T-tests or chi-square tests were used for continuous or categorical variables, respectively. Logistic regression was conducted to explore the association between visual impairment and anxiety symptoms. Covariates included age, gender, residence, marital status, educational level, medical insurance, annual household income, current smoking, current drinking, current exercise, BMI, hypertension, diabetes, and cancer. Similar analyses were conducted to examine the relationships between sleep quality, sleep duration, exercise, DDS, plant DDS, animal DDS, and anxiety. Mediation analyses and Sensitivity analyses were then used to assess the mediating roles of sleep quality, sleep duration, exercise, DDS, plant DDS, and animal DDS in the relationship between visual impairment and anxiety symptoms (28). All statistical analyses were performed using R software version 4.3.3. The R packages used for analysis included “haven,” “dplyr,” “future,” “VIM” and “bruceR.” Statistical tests were two-sided, with significance set at *p* < 0.05.

## 3 Results

### 3.1 Descriptive characteristics

The baseline characteristics of the population in this study was listed in Table 1. After excluding participants under 65 years of age and those with missing values in the exposure variables, mediator variables, and outcome variables, a total of 11,702 samples were included in the study. The age of the study participants ranged from 65 to 117, with an average age of 83.62. Among the participants, 39.4% were aged between 65 and 80 years, 48.6% were aged between 80 and 100 years, and 12.0% were aged over 100 years. In terms of gender, 46.8% were male and 53.2% were female. Regarding residence, 23.7% lived in cities, 32.8% lived in towns, and 43.5% lived in rural areas. Marital status showed that 47.2% were married, while 52.8% were unmarried or divorced. Educational level distribution indicated that 43.5% had no formal education, 36.9% had received education between 1 years and 6 years, 16.0% had received education between 7 years and 12 years, and 3.5% had received higher education for more than 13 years. For medical services, 97.6% had received medical services, while 2.4% had not. Annual household income data showed that 81.3% had an income of less than 100,000 RMB, while 18.7% had an income of 100,000 RMB or more. Smoking status revealed that 16.1% were smokers, and 83.9% were non-smokers. Drinking status indicated that 15.5% consumed alcohol, while 84.5% did not. The average BMI was 23.4 ± 29.3. Regarding hypertension, 48.1% had hypertension, while 51.9% did not. For diabetes, 18.7% had diabetes, while 81.3% did not. Cancer prevalence showed that 3.4% had cancer, while 96.6% did not. Heart disease prevalence indicated that 25.5% had heart disease, while 74.5% did not. Stroke prevalence showed that 19.7% had experienced a stroke, while 80.3% had not. For depression, 50.6% had depression, while 40.7% did not. Visual impairment data revealed that 33.3% had visual impairment, while 66.7% did not. The incidence of anxiety in the overall population was 1,175 per 10,000 individuals, 1,442 per 10,000 among those with visual impairments, and 1,042 per 10,000 among those without visual impairments over the individuals included in our study.

**Table 1:** Baseline characteristics of the population in this study.

Variables	Total sample (*N* = 11,702)	Non-anxiety symptoms (*N* = 10,327)	Anxiety symptoms (*N* = 1,375)	*p*
Age				<0.001
65–80	4,608 (39.4%)	4,030 (39.0%)	578 (42.0%)	
80–100	5,693 (48.6%)	5,047 (48.9%)	646 (47.0%)	
100 +	1,401 (12.0%)	1,250 (12.1%)	151 (11.0%)	
Gender				<0.001
Male	5,482 (46.8%)	4,985 (48.3%)	497 (36.1%)	
Female	6,220 (53.2%)	5,342 (51.7%)	878 (63.9%)	
Residence				<0.001
City	2,771 (23.7%)	2,527 (24.5%)	244 (17.7%)	
Town	3,837 (32.8%)	3,332 (32.3%)	505 (36.7%)	
Rural	5,094 (43.5%)	4,468 (43.3%)	626 (45.5%)	
Marital status				0.033
Married	5,529 (47.2%)	4,917 (47.6%)	612 (44.5%)	
Unmarried or divorced	6,173 (52.8%)	5,410 (52.4%)	763 (55.5%)	
Educational level				<0.001
0	5,088 (43.5%)	4,335 (42.0%)	753 (54.8%)	
1–6	4,322 (36.9%)	3,892 (37.7%)	430 (31.3%)	
7–12	1878 (16.0%)	1725 (16.7%)	153 (11.1%)	
13 +	414 (3.5%)	375 (3.63%)	39 (2.84%)	
Medical service				<0.001
Yes	11,416 (97.6%)	10,129 (98.1%)	1,287 (93.6%)	
No	286 (2.4%)	198 (1.92%)	88 (6.40%)	
Annual household income				<0.001
<100,000 RMB	9,510 (81.3%)	8,331 (80.7%)	1,179 (85.7%)	
≥100,000 RMB	2,192 (18.7%)	1996 (19.3%)	196 (14.3%)	
Smoking status				<0.001
Yes	1883 (16.1%)	1706 (16.5%)	177 (12.9%)	
No	9,819 (83.9%)	8,621 (83.5%)	1,198 (87.1%)	
Drinking status				<0.001
Yes	1812 (15.5%)	1,665 (16.1%)	147 (10.7%)	
No	9,890 (84.5%)	8,662 (83.9%)	1,228 (89.3%)	
BMI	23.4 ± 29.3	23.4 ± 30.6	23.2 ± 17.7	0.742
Hypertension				<0.001
Yes	5,629 (48.1%)	4,905 (47.5%)	724 (52.7%)	
No	6,073 (51.9%)	5,422 (52.5%)	651 (47.3%)	
Diabetes				<0.001
Yes	2,183 (18.7%)	1863 (18.0%)	320 (23.3%)	
No	9,519 (81.3%)	8,464 (82.0%)	1,055 (76.7%)	
Cancer				<0.001
Yes	393 (3.4%)	310 (3.00%)	83 (6.04%)	
No	11,309 (96.6%)	10,017 (97.0%)	1,292 (94.0%)	
Heart disease				<0.001
Yes	2,988 (25.5%)	2,529 (24.5%)	459 (33.4%)	
No	8,714 (74.5%)	7,798 (75.5%)	916 (66.6%)	
Stroke				<0.001
Yes	2,300 (19.7%)	1942 (18.8%)	358 (26.0%)	
No	9,402 (80.3%)	8,385 (81.2%)	1,017 (74.0%)	
Depression				<0.001
Yes	5,917 (50.6%)	5,741 (55.6%)	176 (12.8%)	
No	4,762 (40.7%)	4,586 (44.4%)	176 (12.8%)	
Visual impairment				<0.001
Yes	3,891 (33.3%)	3,330 (32.2%)	561 (40.8%)	
No	7,811 (66.7%)	6,997 (67.8%)	814 (59.2%)	

### 3.2 Association between visual impairment and anxiety symptoms

**Figure 2:**
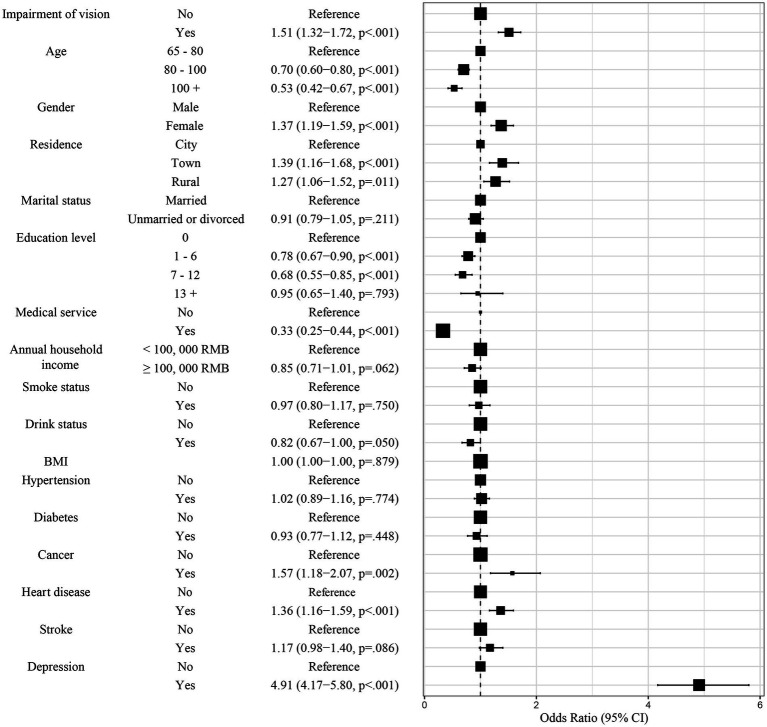
Adjusted ORs from Model 3. CI, Confidence Interval; OR, Odds Ratio.

The association between visual impairment and anxiety symptoms was assessed through model of the binary logistic regression. Model 1 was used to assess the association between visual impairment and anxiety symptoms without adjustment. Compared to those without visual impairment, participants having visual impairment were faced with a significantly increased level of anxiety (OR = 1.72, 95% CI: 1.53–1.94, *p* < 0.001, Table 2 and Supplementary Table 1). Furthermore, Model 2 was used to assess the association between visual impairment and anxiety symptoms with adjustment of several factors, including age, gender, residence, marital status, medical service, annual household income, smoke status, drink status, BMI and education level. After adjustment, participants having visual impairment were faced with a significantly increased level of anxiety (OR = 1.71, 95% CI: 1.51–1.94, *p* < 0.001, Table 2, Supplementary Table 2), compared to those without visual impairment. Finally, more factors, containing age, gender, residence, marital status, medical service, annual household income, smoke status, drink status, BMI, education level, hypertension, diabetes, cancer, heart disease, stroke and depression, were included for assessing the association between visual impairment and anxiety symptoms. After adjustment, participants having visual impairment were faced with a significantly increased level of anxiety (OR = 1.51, 95% CI: 1.32–1.72, *p* < 0.001, Table 2 and Supplementary Table 3), compared to those without visual impairment. These factors indicated that impairment of vision was an essential predictor for anxiety symptoms in older adults in China. The forest plot (Figure 2) indicated the following factors may be likely facilitators for anxiety symptoms for the older adult in China: age, gender, residence, medical service, annual household income, education level, cancer, heart disease and depression.

**Table 2:** Binary logistic regression to estimate the relationship between visual impairment and anxiety symptoms.

Characteristics	Model 1		Model 2		Model 3	
	OR (95%CI)	*p*	OR (95%CI)	*p*	OR (95%CI)	*p*
Without visual impairment	1 (reference)	<0.001	1 (reference)	<0.001	1 (reference)	<0.001
Visual impairment	1.72 (1.53–1.94)		1.71 (1.51–1.94)		1.51 (1.32–1.72)	

### 3.3 The parallel mediation effect model

The mediation effect on the relation between visual impairment and anxiety symptoms was tested through a parallel mediation model. Thus, a bootstrap test was conduct to verify the mediating effect of sleep quality, sleep duration, exercise, DDS, plant DDS and animal DDS. The results indicated that the mediating effects of sleep quality, DDS and plant DDS on the association between visual impairment and anxiety symptoms were significant, while the mediating effects of sleep duration, exercise and animal DDS on the association between visual impairment and anxiety symptoms were not significant. The indirect effect of visual impairment on anxiety symptoms through sleep quality was significant (Effect = 0.0084, 95% CI: 0.0058, 0.0110, *p* < 0.001), accounting for 20.59% of the total effect. The indirect effect of visual impairment on anxiety symptoms through DDS was significant (Effect = 0.0014, 95% CI: 0.0007, 0.0024, *p* < 0.001), accounting for 3.43% of the total effect. The indirect effect of visual impairment on anxiety symptoms through plant DDS was significant (Effect = 0.0023, 95% CI: 0.0012, 0.0035, *p* < 0.001), accounting for 5.64% of the total effect. The results demonstrated that sleep quality, DDS and plant DDS partially mediated the association between visual impairment and anxiety symptoms, while sleep duration, exercise and animal DDS did not mediated the association (*p* > 0.05). The detailed data was exhibited in Table 3.

**Table 3:** Evaluation of standardized effects through the parallel mediation mode.

Mediation pathways	Effect	SE	95% CI		*p*	Percentage
			Lower	Upper		
Sleep quality	0.0084	0.0013	0.0058	0.0110	<0.001	20.59%
Sleep duration	0.0004	0.0009	−0.0014	0.0023	0.66	
Exercise	0.0008	0.0008	−0.0007	0.0025	0.27	
DDS	0.0014	0.0004	0.0007	0.0024	<0.001	3.43%
Plant DDS	0.0023	0.0005	0.0012	0.0035	<0.001	5.64%
Animal DDS	0.0002	0.0002	−0.0001	0.0005	0.29	

**Figure 3:**
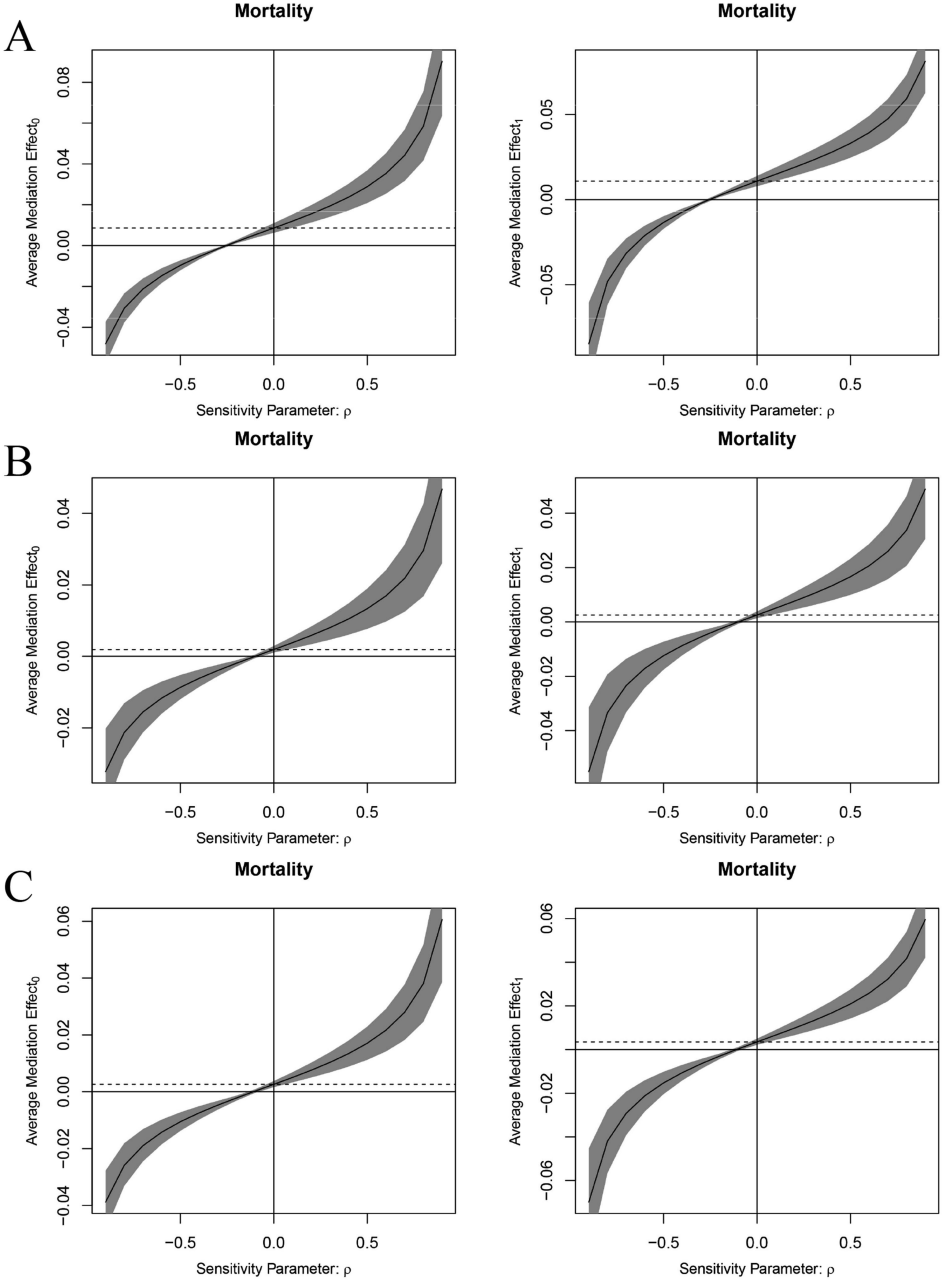
Sensitivity analyses for mediating effect of sleep quality **(A)**, DDS **(B)**, plant-based DDS **(C)**.

Sensitivity analyses were conduct to evaluate the potential influence of unobserved confounders on mediation effects, respectively. For mediating effect of sleep quality, our sensitivity analysis revealed that the correlation coefficient (*ρ*) of unobserved confounding factors must reach-0.3 for the Average Causal Mediation Effect (ACME) to reduce to zero (Figure 3A). Further analysis indicated that these unobserved confounders need to account for 9% of the variance between the mediator and the outcome variable, or explain 7.28% of the variance, to completely negate the mediation effect. These findings underscore the robustness of sleep quality as a mediator between visual impairment and anxiety, demonstrating that the mediation effect remains significant even when considering potential unobserved confounding factors. For mediating effect of DDS, our sensitivity analysis revealed that the ACME reduces to zero when the correlation coefficient (*ρ*) of unobserved confounders reaches-0.1 (Figure 3B). Additionally, these confounders need to explain 1% of the variance between the mediator and the outcome variable or 0.0075 to completely negate the mediation effect. These findings suggest that despite the presence of unobserved confounders, the mediation effect of DDS demonstrates relative robustness. For mediating effect of plant-based DDS, our sensitivity analysis revealed that the ACME reduces to zero when the correlation coefficient (ρ) of unobserved confounders reaches-0.1 (Figure 3C). Additionally, these confounders need to explain 1% of the variance between the mediator and the outcome variable or 0.0078 to completely negate the mediation effect. These findings suggest that despite the presence of unobserved confounders, the mediation effect of DDS demonstrates relative robustness.

## 4 Discussion

This study aimed to investigate the association between vision impairment and anxiety symptoms among the Chinese older adult. Additionally, it evaluated the potential effects and mediating roles of sleep quality, sleep duration, exercise, Dietary Diversity Score (DDS), plant-based DDS, and animal-based DDS in this relationship. Consistent with our previous hypothesis, the Chinese older adult individuals with vision impairment were more likely to experience anxiety. Sleep quality, DDS, and plant-based DDS appeared to have parallel mediating roles in the association between vision impairment and anxiety symptoms, while sleep duration, exercise and animal-based DDS did not demonstrate significant mediating effects.

Compared to physical problems, the psychological issues of the older adult have long been neglected. However, in recent years, the psychological problems of the older adult have begun to receive increasing attention from researchers (29–31). According to research reports, various factors can lead to anxiety symptoms in the older adult, such as chronic disease (32), financial strain (33), depression (34), and so on. This study found that visual impairment is one of the risk factors for anxiety symptoms, which is consistent with previous studies (3, 6, 35, 36). Moreover, this study also shows that the incidence of anxiety symptoms is higher among participants with visual impairment than those without. All these findings suggest that attention and care should be given to the anxiety symptoms of older adult people with visual impairment to improve their quality of life.

Furthermore, our study explored the mediating effects of several factors on the relationship between visual impairment and anxiety symptoms through a parallel mediation model. Sleep quality act as a significant tole of in the lives of the Chinese older adult with visual impairment (37). Older adult individuals with visual impairments may experience disrupted sleep patterns due to difficulties in maintaining a regular sleep–wake cycle (38), increased physical discomfort, and stress associated with their condition. These sleep disturbances can exacerbate anxiety symptoms, creating a vicious cycle (39). Poor sleep quality can lead to increased irritability (40, 41), reduced cognitive function (42), and a diminished ability to cope with stress (43, 44), all of which can heighten anxiety (45–47). Conversely, improving sleep quality can enhance mood, cognitive function, and stress resilience, potentially mitigating anxiety symptoms. These findings underscore the importance of addressing sleep quality as a therapeutic target in this population. In contrast, sleep duration did not show significant mediating effects. These results are aligned with previous research indicating that sleep quality, rather than sleep duration, is more closely associated with mental health outcomes. Simply extending sleep duration has no significant effect on relieving anxiety symptoms.

As for plant diet diversity, plant diet diversity refers to the variety of plant-based foods consumed, which can significantly impact overall health and well-being (48, 49). A diverse plant diet provides essential nutrients, antioxidants, and anti-inflammatory compounds that are crucial for maintaining physical and mental health (50, 51). For older adult individuals with visual impairments, maintaining a diverse diet can be challenging due to difficulties in food preparation and access to a variety of foods (52, 53). A diverse diet rich in vegetables, fruits, legumes, nuts, garlic and mushrooms can improve gut health, reduce inflammation, and support brain function, all of which are associated with reduced anxiety (54). Conversely, a lack of dietary diversity can lead to nutritional deficiencies and poorer health outcomes, exacerbating anxiety symptoms (55). Specific nutrients found in a diverse plant-based diet, such as omega-3 fatty acids (55), vitamins (56), and minerals (57, 58), are known to influence mood and cognitive function. These findings underscore the importance of promoting dietary diversity as a strategy to alleviate anxiety symptoms in individuals with visual impairments. In contrast, animal-based DDS did not show significant mediating effects.

Exercise did not show significant mediating effects, as well. This suggests that while exercise is important for general health, they may not play a pivotal role in the specific pathway between visual impairment and anxiety symptoms.

These findings are multifaceted and could benefit the Chinese older adult with visual impairment. First, they highlight the necessity of integrating vision care into broader health and wellness programs for the older adult. Regular eye check-ups and interventions to prevent or mitigate visual impairment could be crucial steps in reducing anxiety and improving overall mental health. Second, improving sleep quality through interventions may be beneficial for older adult individuals with visual impairment. Third, promoting dietary diversity, particularly plant-based diets, could serve as an additional strategy to enhance mental well-being in this population. Future research should continue to explore the underlying mechanisms through which visual impairment influences anxiety and other mental health outcomes. Longitudinal studies could provide more insights into causal relationships and the effectiveness of various interventions. Additionally, investigating the role of other potential mediators, such as social support and physical activity, could further elucidate the complex interplay between visual impairment and mental health. However, it is essential to recognize certain limitations in this study. Firstly, the dataset used in this study was derived from a 2018 survey, which may not comprehensively reflect current trends or shifts in health behaviors and social dynamics within the older adult population in China. As social and healthcare practices evolve, the generalizability of the findings to present-day contexts may be limited. Secondly, the reliance on self-reported data introduces potential biases, such as recall bias or social desirability bias, which could compromise the accuracy of reported information regarding visual impairment, sleep quality, dietary diversity, and anxiety symptoms. Future studies would benefit from the inclusion of objective measures for these variables, as well as the use of more recent datasets, to enhance the robustness and generalizability of the conclusions.

## 5 Conclusion

This study investigates the association between visual impairment and anxiety symptoms in the Chinese older adult. After adjusting for multiple covariates, the anxiety symptoms of older adult with visual impairment individuals remain significantly higher. The results of parallel mediation analysis indicate that sleep quality, DDS, and plant DDS have parallel mediation effects. Sleep quality, DDS and plant DDS may explain the pathway from visual impairment to anxiety symptoms, suggesting that intervention strategies for anxiety symptoms in Chinese older adult individuals with visual impairment should be different. Poor sleep quality and a monotonous diet diversity, particularly in plant-based diet, are positively correlated with anxiety symptoms in Chinese older adult individuals with visual impairment. It is noteworthy that merely extending sleep duration or increasing dietary diversity based on animal sources cannot effectively alleviate the anxiety symptoms of Chinese older adult individuals with visual impairment. Therefore, appropriate interventions should be implemented to reduce the anxiety symptoms of Chinese older adult individuals with visual impairment, encouraging them to improve sleep quality and increase dietary diversity, especially the diversity of plant-based foods. Future research could explore the specific types of plant-based foods that most effectively contribute to reducing anxiety symptoms, as well as the role of other dietary components such as micronutrients in this context. Additionally, longitudinal studies are needed to examine whether sustained improvements in sleep quality and dietary diversity can have long-term effects on reducing anxiety. Investigating potential cultural or socioeconomic factors that influence dietary and sleep behaviors in this population could also provide more comprehensive insights for targeted interventions.

## Data Availability

The datasets presented in this study can be found in online repositories. The names of the repository/repositories and accession number(s) can be found at: https://chads.nsd.pku.edu.cn/sjzx/index.htm.
