# Supplementary material for: Were there any missing mediators between visual impairment and anxiety symptoms? Results from Chinese Longitudinal Healthy Longevity Survey
Source: Front Public Health. 2024 Oct 16;12:1448638. doi: 10.3389/fpubh.2024.1448638 (PMC11521831; doi:10.3389/fpubh.2024.1448638)
Supplement: Supplementary file 1 [file Table_1.DOCX]

Supplementary table 1. Binary logistic regression to estimate the association between impairment of vision and anxiety symptoms (Model 1)

| Characteristics | | *P* | OR | (95% CI) |
| --- | --- | --- | --- | --- |
| Impairment of vision | No |  | 1 | Reference |
|  | Yes | *P* < 0.001 | 1.72 | (1.53-1.94) |

OR: Odds Ratio. 95%CI: 95% Confidence Interval. Model 1: without adjustment.

Supplementary table 2. Binary logistic regression to estimate the association between impairment of vision and anxiety symptoms (Model 2)

| Characteristics | | *P* | OR | (95% CI) |
| --- | --- | --- | --- | --- |
| Impairment of vision | No |  | 1 | Reference |
|  | Yes | *P* < 0.001 | 1.71 | (1.51-1.94) |
| Age | 65 - 80 |  | 1 | Reference |
|  | 80 - 100 | *P* < 0.001 | 0.68 | (0.59-0.79) |
|  | 100 + | *P* < 0.001 | 0.49 | (0.39-0.62) |
| Gender | Male |  | 1 | Reference |
|  | Female | *P* < 0.001 | 1.36 | (1.18-1.56) |
| Residence | City |  | 1 | Reference |
|  | Town | 0.002 | 1.32 | (1.11-1.58) |
|  | Rural | 0.094 | 1.16 | (0.97-1.39) |
| Marital status | Married |  | 1 | Reference |
|  | Unmarried or divorced | 0.907 | 0.99 | (0.86-1.14) |
| Medical service | No |  | 1 | Reference |
|  | Yes | *P* < 0.001 | 0.31 | (0.24-0.40) |
| Annual household income | < 100,000 RMB |  | 1 | Reference |
|  | ≥ 100,000 RMB | 0.026 | 0.83 | (0.70-0.98) |
| Smoke status | No |  | 1 | Reference |
|  | Yes | 0.636 | 0.96 | (0.79-1.15) |
| Drink status | No |  | 1 | Reference |
|  | Yes | 0.002 | 0.74 | (0.61-0.90) |
| BMI | Mean ± SD | 0.953 | 1.00 | (1.00-1.00) |
| Education level | 0 |  | 1 | Reference |
|  | 1-6 | *P* < 0.001 | 0.73 | (0.64-0.85) |
|  | 6-12 | *P* < 0.001 | 0.63 | (0.51-0.79) |
|  | 13+ | 0.547 | 0.89 | (0.61-1.30) |

OR: Odds Ratio. 95%CI: 95% Confidence Interval. Model 1: adjusted for age, gender, residence, marital status, medical service, annual household income, smoke status, drink status, BMI and education level.

Supplementary table 3. Binary logistic regression to estimate the association between impairment of vision and anxiety symptoms (Model 3)

| Characteristics | | *P* | OR | (95% CI) |
| --- | --- | --- | --- | --- |
| Impairment of vision | No |  | 1 | Reference |
|  | Yes | *P* < 0.001 | 1.51 | (1.32-1.72) |
| Age | 65 - 80 |  | 1 | Reference |
|  | 80 - 100 | *P* < 0.001 | 0.70 | (0.60-0.80) |
|  | 100 + | *P* < 0.001 | 0.53 | (0.42-0.67) |
| Gender | Male |  | 1 | Reference |
|  | Female | *P* < 0.001 | 1.39 | (1.19-1.59) |
| Residence | City |  | 1 | Reference |
|  | Town | *P* < 0.001 | 1.39 | (1.16-1.68) |
|  | Rural | 0.011 | 1.27 | (1.06-1.52) |
| Marital status | Married |  | 1 | Reference |
|  | Unmarried or divorced | 0.211 | 0.91 | (0.79-1.05) |
| Medical service | No |  | 1 | Reference |
|  | Yes | *P* < 0.001 | 0.33 | (0.25-0.44) |
| Annual household income | < 100,000 RMB |  | 1 | Reference |
|  | ≥ 100,000 RMB | 0.062 | 0.83 | (0.71-1.01) |
| Smoke status | No |  | 1 | Reference |
|  | Yes | 0.750 | 0.97 | (0.80-1.17) |
| Drink status | No |  | 1 | Reference |
|  | Yes | 0.050 | 0.82 | (0.67-1.00) |
| BMI | Mean ± SD | 0.879 | 1.00 | (1.00-1.00) |
| Education level | 0 |  | 1 | Reference |
|  | 1-6 | *P* < 0.001 | 0.78 | (0.67-0.90) |
|  | 6-12 | *P* < 0.001 | 0.68 | (0.55-0.85) |
|  | 13+ | 0.793 | 0.95 | (0.65-1.40) |
| Hypertension | No |  | 1 | Reference |
|  | Yes | 0.774 | 1.02 | 0.89-1.16 |
| Diabetes | No |  | 1 | Reference |
|  | Yes | 0.448 | 0.93 | (0.77-1.12) |
| Cancer | No |  | 1 | Reference |
|  | Yes | 0.002 | 1.57 | (1.18-2.07) |
| Heart disease | No |  | 1 | Reference |
|  | Yes | *P* < 0.001 | 1.36 | (1.16-1.59) |
| Stroke | No |  | 1 | Reference |
|  | Yes | 0.086 | 1.17 | (0.98-1.40) |
| Depression | No |  | 1 | Reference |
|  | Yes | *P* < 0.001 | 4.91 | (4.17-5.80) |

OR: Odds Ratio. 95%CI: 95% Confidence Interval. Model 1: adjusted for age, gender, residence, marital status, medical service, annual household income, smoke status, drink status, BMI, education level, hypertension, diabetes, cancer, heart disease, stroke and depression
